# Cyclosporine as a Novel Therapeutic Option for Eosinophilic Cystitis in a Paraplegic Dog

**DOI:** 10.1155/crve/7317611

**Published:** 2026-07-13

**Authors:** Lara Vilela Soares, Yury Carantino Costa Andrade, Maria Eduarda Raffaini de Oliveira Cunha, Alessandra Aparecida Medeiros Ronchi, Vanessa Martins Fayad Milken, Sofia Borin-Crivellenti, Leandro Z. Crivellenti

**Affiliations:** ^1^ Graduate Program in Veterinary Science (PPGCV), School of Veterinary Medicine and Animal Science, Federal University of Uberlândia (UFU), Uberlândia, Minas Gerais, Brazil, ufu.br

**Keywords:** chronic cystitis, cyclosporine, eosinophilic inflammation, spinal cord injury

## Abstract

Eosinophilic cystitis is characterized by eosinophilic infiltration and proliferation within the layers of the bladder wall. Its etiology remains unclear, and there is no established consensus regarding treatment in either human or veterinary medicine. A 4‐year‐old female dog developed paraplegia after a car accident, with a spinal cord injury in the lumbar region diagnosed by radiography. Five months after the trauma, the animal began to present with hematuria and recurrent episodes of urinary tract infection, with an unsatisfactory response to conventional therapies with bladder emptying management and antibiotics for 1 year. Bladder wall biopsy revealed findings consistent with eosinophilic cystitis. Due to the persistence of clinical signs, cyclosporine treatment was initiated, alongside previous surgery and antibiotics, resulting in significant clinical improvement, normalization of bladder ultrasonographic findings, and no observed adverse effects during the treatment period. The medication was continued for 6 months, followed by a gradual tapering. Six months after discontinuation, the animal remained asymptomatic. This case highlights the potential use of cyclosporine in the treatment of eosinophilic cystitis and supports the hypothesis that spinal cord injury may represent a possible risk factor for EC in dogs. It also emphasizes the importance of including EC in the differential diagnosis of bladder wall thickening. This report has some limitations, including the evaluation of a single patient, the use of concurrent therapies, and the absence of serum cyclosporine level monitoring.

**Summary**

• Eosinophilic cystitis should be considered in dogs presenting with gross hematuria, dysuria, and bladder wall thickening.
• Histopathology remains the gold standard for a definitive diagnosis.
• Cyclosporine can be considered a therapeutic option for eosinophilic cystitis, especially in refractory cases.

## 1. Background

Eosinophilic cystitis (EC) is considered an immune‐mediated inflammatory condition characterized by eosinophilic infiltration of the bladder wall, often associated with hypersensitivity reactions and chronic antigenic stimulation [1, 2]. Evidence suggests that eosinophil activation in this condition is mediated by T lymphocytes and cytokines, particularly interleukin (IL)‐5, which plays a central role in eosinophil recruitment, activation, and survival [2].

The etiology of EC remains under investigation in both veterinary and human medicine. In dogs, the condition has been associated with a history of urolithiasis, chronic inflammation of the urinary tract, and the presence of polypoid bladder masses [3, 4]. In human medicine, the most relevant findings that may contribute to veterinary understanding of EC include the proposed role of immune dysregulation and hypersensitivity‐mediated inflammation, characterized by eosinophilic infiltration associated with T‐cell activation and increased expression of certain cytokines [5]. These mechanisms may help explain the chronic inflammatory response observed in canine cases and support the use of immunomodulatory therapies. Additionally, human reports describing EC as a condition capable of mimicking bladder neoplasia on imaging and cystoscopic evaluation are particularly relevant to veterinary medicine, as canine patients may also present with bladder wall thickening, mass‐like lesions, or polypoid proliferations that complicate the differential diagnosis with transitional cell carcinoma and other urinary bladder tumors [6, 1]. Therefore, observations from human medicine may provide useful translational insight into the diagnostic challenges, immunopathogenesis, and therapeutic strategies applicable to canine EC.

Symptoms such as urinary urgency, dysuria, and gross hematuria are commonly reported in both human and canine patients [7, 8]. Symptoms such as urinary urgency, dysuria, and gross hematuria are commonly reported in both human and canine patients [9]. Histopathological evaluation remains the gold standard for the diagnosis of EC, which is characterized by diffuse and often transmural inflammatory infiltration of the urinary bladder wall, predominantly composed of eosinophils, and frequently associated with edema, fibrosis, and variable numbers of lymphocytes and plasma cells [10]. In some cases, increased local immunoglobulin expression, particularly IgE and IgA, has also been described, supporting the hypothesis of an immune‐mediated or hypersensitivity‐associated inflammatory process [11].

There is no established consensus on treatment in humans, and most studies initially focus on conservative approaches, including antihistamines, corticosteroids, and monoclonal antibodies [12, 13]. In veterinary medicine, reported cases are scarce, and treatment protocols generally focus on urolith removal, partial cystectomy, and administration of corticosteroids or other immunosuppressive agents. Corticosteroid therapy has been described as the most frequently used medical approach in dogs, with reports of marked clinical improvement and resolution of urinary abnormalities following prednisone administration combined with management of potential hypersensitivity triggers, such as dietary modification and antiparasitic therapy [14]. However, information regarding alternative immunosuppressive protocols, long‐term clinical outcomes, and therapeutic response in refractory or recurrent cases remains limited in veterinary medicine, highlighting the clinical relevance of the present report.

Although the exact immunopathogenesis of EC remains unclear, eosinophilic infiltration is believed to result from an exaggerated immune response mediated by T lymphocytes and the release of proinflammatory cytokines, particularly those associated with Th2‐type responses, such as IL‐4, IL‐5, and IL‐13. These cytokines promote eosinophil recruitment, activation, and persistence within the bladder wall, contributing to chronic inflammation and tissue damage. Cyclosporine is a calcineurin inhibitor with immunomodulatory effects that suppresses T‐cell activation and cytokine production by inhibiting IL‐2 transcription. Through this mechanism, cyclosporine may reduce eosinophilic inflammation and interrupt the immune‐mediated cascade involved in EC pathogenesis. In veterinary medicine, cyclosporine is primarily used for canine atopic dermatitis, perianal fistulas, sebaceous adenitis, and other immune‐mediated or autoimmune diseases, often as a steroid‐sparing drug for long‐term management [15]. Adverse effects in dogs are generally uncommon, with gastrointestinal signs such as vomiting, diarrhea, lethargy, and anorexia being the most frequently reported; less common effects include pruritus, gingival hyperplasia, and increased liver enzyme activity. Overall, most adverse effects are mild and improve with dose adjustment, and cyclosporine is considered relatively safe for long‐term use when appropriately monitored [16].

To our knowledge, this is the first reported case of EC in a paraplegic dog and one of the few veterinary reports describing the use of cyclosporine as part of the therapeutic management [8]. Unlike the previously reported canine case treated with cyclosporine, the present report describes EC in a paraplegic dog with chronic neurologic dysfunction and impaired urinary bladder function, factors that may have contributed to persistent lower urinary tract inflammation and diagnostic complexity. Therefore, this case expands the current veterinary literature by highlighting a distinct clinical presentation and supporting the potential role of cyclosporine in complex or refractory cases of EC.

## 2. Case Presentation

A 4‐year‐old spayed female mixed‐breed dog, weighing 7.8 kg, with a history of paraplegia secondary to a traumatic spinal cord injury (SCI) caused by an automobile accident, diagnosed via radiographic evaluation as a spinal fracture affecting the T3–L1 region, presented with persistent (macroscopic) hematuria and a strong‐smelling urine of several months′ duration that was unresponsive to antibiotics. Advanced imaging modalities, such as computed tomography (CT) or magnetic resonance imaging (MRI), were not performed due to financial limitations. Clinical signs had been present for more than 6 months prior to presentation. Prior treatments included amoxicillin–clavulanate and enrofloxacin, each administered for 10 days, with an interval of approximately 30 days between therapies; however, treatment was not guided by urine culture and antimicrobial susceptibility testing. Bladder emptying was managed by manual expression performed three times daily since onset of paraplegia, and no intermittent or indwelling urinary catheterization had been performed before referral. Due to the dog′s paraplegia, it was not possible to assess dysuria or urinary urgency. On physical examination, the patient exhibited paraplegia, no response to painful stimuli, and marked disuse muscle atrophy, with a body condition score of 3/9. Neurologic examination revealed paraplegia with absent nociception in the pelvic limbs, findings consistent with an upper motor neuron (UMN) lesion affecting the T3–L3 spinal cord segments, in accordance with the location of the vertebral injury (T3–L1). Anal tone and perineal reflexes were intact, indicating preserved lower motor neuron (L4–S3) function.

Laboratory tests, including a complete blood count and initial serum biochemistry, were within normal limits for the species. Urinalysis and urine culture with antibiogram revealed a urine‐specific gravity of 1.033, pH 8, pyuria, red blood cells, and a significant presence of bacteria (++++). Bacterial identification was performed using culture and biochemical tests: Gram‐positive bacteria were assessed with catalase, oxidase, mannitol fermentation, coagulase, and bile tests, whereas Gram‐negative bacteria were identified using the Bactray I, II, and III systems. Antimicrobial susceptibility testing was conducted according to standard protocols. The culture showed growth of two bacterial colonies, *Staphylococcus* spp. and *Proteus penneri*, both displaying sensitivity to amoxicillin + clavulanate, amikacin, and florfenicol (Figure 1). All urine samples were collected using ultrasound‐guided cystocentesis.

**Figure 1:**
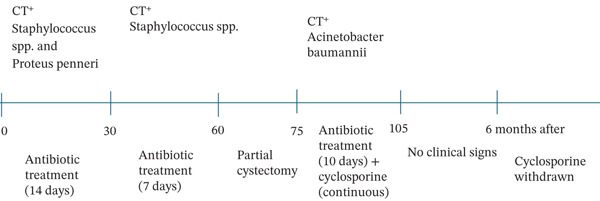
Treatment timeline in days. Chronological representation of the patient′s clinical course, including antibiotics and cyclosporine treatment. CT+: positive culture.

Abdominal ultrasound was performed and demonstrated a urinary bladder with preserved contours but marked wall thickening, mild irregularity, and an anechoic content, and sediment adhered to the mucosa and deposited within the bladder lumen (Figure 2A). The patient was treated with amoxicillin + clavulanate (20 mg/kg, orally, q12h for 14 days). Figure 2

**Figure 2:**
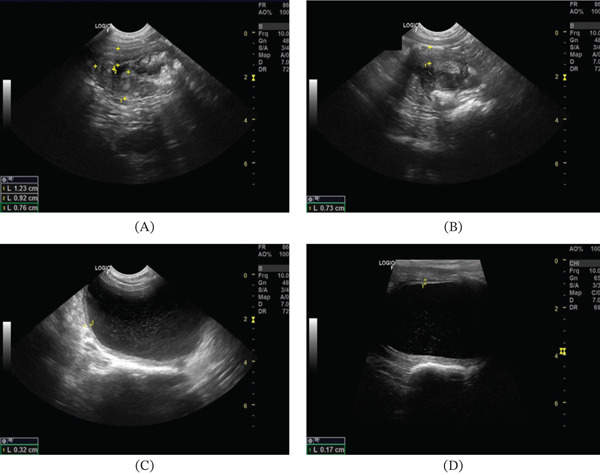
Ultrasonographic evaluation of the bladder in B‐mode at different stages of treatment. (A) Ultrasound image of the bladder obtained in the initial clinical evaluation. Topographically normal urinary bladder with preserved contour, slightly irregular and thickened wall in dorsal (±1.23 cm) and ventral portion (±0.76 cm). Anechoic content with a large amount of hyperechoic sediment adhered and deposited in the lumen. (B) Postcystectomy ultrasonographic image of the bladder showing a mildly irregular and thickened wall (±0.73 cm), with minimal anechoic content and echogenic and hyperechoic sediment suspended and adhered to the mucosa of the bladder. (C) Ultrasonography 1 month after cyclosporine treatment. Topographically normal urinary bladder with moderate filling, preserved contour, regular and mildly thickened wall (±0.32 cm), anechoic content with moderate suspended echoes. (D) Ultrasonography 6 months after withdrawing the cyclosporine showing regular and thin‐walled (±0.17 cm) anechoic content with suspended echoes (urinary sediment) in moderate quantity.

**Figure 3:**
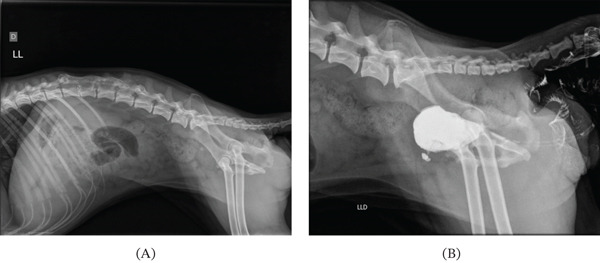
Imaging findings of the thoracolumbar spine and the urinary bladder. (A) Lateral radiograph of the thoracolumbar spine showing intervertebral foraminal space narrowing at L1–L2, along with bony remodeling of the articular processes at T13–L4, as well as of the vertebral arch laminae of L1–L3, suggesting ongoing consolidation of a previous vertebral fracture. (B) Cystogram demonstrating a reduced and abnormally shaped urinary bladder with irregular, thickened wall contours and a contrast‐filling defect. A contrast column adjacent to the cranioventral bladder wall is visible and communicates with the bladder wall, measuring approximately 0.85 × 0.37 cm. A thin line of contrast was also visible within the urethra. These findings are consistent with chronic inflammatory changes of the urinary bladder.

**Figure 4:**
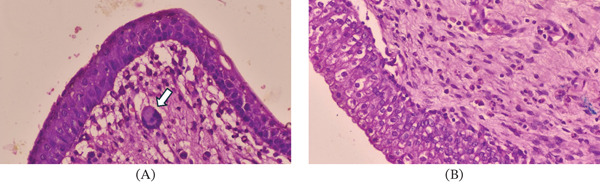
Histopathological features of the bladder. (A) Thickening of the lamina propria with numerous capillaries filled with erythrocytes. Urothelial cells invaginating into the lamina propria, consistent with Brunn nests (white arrow). Microscopic view (objective lens 20x). (B) Urothelial hyperplasia and inflammatory infiltrate composed of neutrophils and lymphocytes. Microscopic view (objective lens 40x). Stained with hematoxylin and eosin (H&E).

One month later, the dog returned to the hospital with dark brown urine, blood clots, and a foul odor. A new urinalysis showed a urine‐specific gravity of 1.042, pH 8, pyuria, hematuria, and the presence of bacteria (4+), and the culture was positive for *Streptococcus* spp. sensitive only to nitrofurantoin. Positive contrast radiographs revealed thickening and irregular contours of the bladder wall (Figure 3B), findings suggestive of ongoing chronic inflammatory changes.

Initial treatment included meloxicam (0.1 mg/kg, orally, q24h for 5 days) and nitrofurantoin (2.5 mg/kg, orally, q6h for 7 days). Due to persistent treatment refractoriness, hematuria, and a contrast‐filling defect visibly communicating with the bladder wall, a partial cystectomy of the affected region was performed. During surgery, the bladder wall showed marked thickening, and a 1.5 cm full‐thickness fragment, including the mucosa, submucosa, and muscular layer, was collected, fixed in 10% neutral buffered formalin, routinely processed, paraffin‐embedded, sectioned, and stained with hematoxylin and eosin (H&E) for histopathological evaluation.

Following surgery, persistent hematuria was observed, which was considered an expected finding during the immediate postoperative period due to surgical manipulation and associated inflammation. Ultrasonography 11 days postcystectomy (Figure 2B) revealed bladder wall changes consistent with chronic cystitis. Despite the persistent hematuria, the patient maintained a normal appetite, defecation, and hematological parameters.

Histopathological evaluation revealed urothelial hyperplasia, with areas of mucosal ulceration and a marked inflammatory infiltrate composed predominantly of eosinophils, with fewer neutrophils (Figure 4A). The lamina propria was thickened due to abundant loose connective tissue and exhibited numerous capillaries filled with erythrocytes (Figure 3B). Occasionally, structures formed by urothelial cells invaginating into the lamina propria were observed, consistent with Brunn nests. The diagnosis was confirmed by two independent veterinary pathologists as EC.

After the partial cystectomy, the patient continued to present with hematuria with blood clots. Therefore, a repeat urine culture and antimicrobial susceptibility test identified *Acinetobacter baumannii*, sensitive to amikacin, ceftriaxone, and nitrofurantoin. Given the persistence of clinical signs despite previous antimicrobial therapies, the chronicity of the condition, and histopathological findings consistent with eosinophilic inflammation, EC was considered at this stage. Based on these findings, treatment was initiated with nitrofurantoin (2.5 mg/kg, orally, q6h for 10 days) and cyclosporine (5 mg/kg, q24h, for continuous use), aiming to address both the bacterial infection and the suspected immune‐mediated component.

## 3. Outcome and Follow‐Up

Within 72 h of initiating cyclosporine, macroscopic hematuria resolved, and it remained resolved after 1 month of therapy. In addition to the absence of abnormalities on physical examination, laboratory tests remained within reference values, including leukocytes (13.6 × 10^3^/*μ*L; ref: 6.0–17.0 × 10^3^/*μ*L), eosinophils (1088/*μ*L; ref: 150–1250/*μ*L), creatinine (1.08 mg/dL), and urea (24.9 mg/dL). One month after clinical improvement, the owner discontinued cyclosporine administration for 3 days, after which hematuria recurred on the 4th day. Cyclosporine was continued for 6 months, and the patient was monitored monthly through physical examination, blood count, biochemistry, and urinalysis. During this period, the dog remained free of hematuria, and follow‐up ultrasonography showed normal bladder wall thickness and contour, without adverse effects from the medication. Ultrasound showed substantial improvement in bladder wall appearance (Figure 2C). Cyclosporine dose was gradually tapered by reducing the dose by 25% every 2 weeks until complete discontinuation (100% tapering) was achieved. After 6 months without cyclosporine, the dog continued to show no recurrence of hematuria, and CBC and biochemistry remained within normal ranges (Figure 2D). Although urinalysis still showed the presence of bacteria and leukocytes, these were observed in lower numbers, and in the absence of clinical signs, this finding was interpreted as subclinical bacteriuria. Therefore, antimicrobial therapy was not instituted in order to avoid the development of antimicrobial resistance.

## 4. Discussion

EC is rare in both human and veterinary medicine, with most reported cases in dogs associated with a history of urolithiasis [3, 8, 17]. However, in the present case, no uroliths were identified, and there was no prior history. Instead, the dog had a history of spinal cord trauma, which could represent a potential risk factor for neurogenic bladder dysfunction and incomplete urine emptying. While no cases directly linking paraplegia to EC have been reported, studies have shown that SCI increases the risk of urinary tract infections due to incomplete urine elimination and biofilm formation [18], which may in turn contribute to chronic bladder inflammation [19].

A murine study [20] demonstrated that SCI leads to focal loss of umbrella cells in the urothelium and the emergence of abnormally differentiated superficial cells coexpressing basal and intermediate keratins, indicating an impaired regenerative process and persistent urothelial immaturity. Additionally, studies on bladder biopsies from SCI patients, even in the absence of urinary symptoms, revealed that 91.5% exhibited histopathological alterations and approximately 67% showed evidence of chronic inflammation [21]. Another study reported increased expression of inflammatory proteins such as tryptase, TNF‐*α*, and TGF‐*β* [14]. These findings suggest that chronic bladder inflammation secondary to spinal injury can alter urothelial homeostasis. While no causal relationship can be established with EC, it is worth considering whether similar mechanisms might play a role in this case. To our knowledge, this is the first report to raise this hypothesis in veterinary literature.

The diagnosis of EC can be challenging due to its clinical and imaging similarities with bladder neoplasia. Both conditions may present with bladder wall thickening, hematuria (macroscopic or microscopic), and dysuria [1, 22]. In human medicine, reports describe the imaging characteristics of EC lesions as a tumor‐like mass, mimicking bladder cancer [23, 24]. A similar diagnostic overlap occurs in veterinary medicine. In a reported case of canine EC, urine cytology revealed squamous cells with atypical urothelial features and no eosinophiluria, initially raising suspicion of malignancy [8]. However, histopathological evaluation of the bladder wall confirmed the diagnosis of EC. Similarly, in our case, the definitive diagnosis was also established through histopathology, underscoring the role of tissue biopsy in distinguishing EC from malignancy. Thus, histopathology remains the gold standard for definitive diagnosis.

Ultrasound and urography are considered nonspecific for definitive diagnosis, but ultrasound can be useful for monitoring the therapeutic response, as evidenced by improvement in the appearance of the bladder wall. This is consistent with reports that recommend ultrasound as the imaging modality of choice for follow‐up evaluations [25]. In the present case, this exam played an active role in monitoring the clinical course and remission of the animal′s bladder lesions.

Regarding treatment, there are no established consensus guidelines for EC; however, reports in human literature suggest the efficacy of corticosteroids (prednisolone 40 mg once daily), antihistamines (clemastine 1 mg twice daily), anti‐inflammatories (diclofenac 50 mg three times daily), and antibiotics in cases with positive cultures for managing the disease [16, 11, 26]. In a retrospective study involving 22 human patients with EC, resection of the tumor‐like mass was the predominant treatment approach, and the concomitant use of corticosteroids led to complete or partial clinical improvement in 72.7% of cases [9]. Another study, involving a pooled analysis of 135 EC cases, showed that treatment effectiveness varied according to mass resection and the use of pharmacologic therapies [2]. Although these findings originate from human medicine, they are presented to provide supportive insights into potential mechanisms and therapeutic approaches, rather than implying direct equivalence, given the limited data available in veterinary patients.

Recently, in humans, other treatment options involving IL inhibition, such as cyclosporine, have been used with good response in EC patients who do not respond to standard therapy. One woman achieved complete symptom resolution with cyclosporine at a dose of 2.3 mg/kg/day, and sustained remission was maintained for 18 months following gradual tapering [27]. Another patient, who presented with multiple comorbidities, was able to reduce corticosteroid dosage and maintain clinical control with cyclosporine use [16]. The use of cyclosporine (7.5 mg/kg, q12) proved to be effective and well‐tolerated in a dog diagnosed with EC and concurrent diabetes mellitus, a condition in which corticosteroid use is contraindicated [8].

EC is considered an immune‐mediated inflammatory condition characterized by eosinophilic infiltration of the bladder wall, frequently associated with hypersensitivity reactions and chronic antigenic stimulation [1, 15]. Evidence suggests that eosinophil activation in this condition is mediated by T lymphocytes and cytokines, particularly IL‐5, which plays a central role in eosinophil recruitment, activation, and survival [15]. Cyclosporine is a calcineurin inhibitor with immunomodulatory effects that suppresses T‐cell activation and cytokine production, thus reducing the eosinophil‐mediated inflammatory response. Therefore, the T‐cell‐targeted immunomodulatory effects of cyclosporine may be particularly suitable for the management of eosinophilic, likely immune‐mediated cystitis. Furthermore, cyclosporine may act to spare the use of steroids to avoid adverse effects associated with prolonged corticosteroid therapy. Therefore, the administration of cyclosporine in this canine patient was based on the immune‐mediated pathophysiology of EC and the need for long‐term immunomodulatory therapy to control chronic bladder inflammation and prevent recurrence of clinical signs. To the authors′ knowledge, this is the first reported canine case of EC treated with cyclosporine in a paraplegic dog with possible neurogenic bladder dysfunction and recurrent urinary tract infections.

In the present report, treatment with cyclosporine (5 mg/kg/day) resulted in a rapid and sustained resolution of hematuria, as similarly reported, along with normalization of inflammatory markers and marked improvement on ultrasonographic examination. The dog remained free of clinical signs throughout the 6‐month treatment period and showed no recurrence after gradual tapering and discontinuation of the medication.

EC should be considered a differential diagnosis in animals presenting with gross hematuria, dysuria, and a thickened bladder wall, especially those with SCI. Cytology may have limited diagnostic value in inflammatory bladder diseases, as it can fail to detect eosinophilic infiltration and may yield nonspecific or misleading findings. Biopsy remains the gold standard for definite diagnosis. EC should be considered a differential diagnosis in animals presenting with macroscopic hematuria, dysuria, and bladder wall thickening, especially those with SCI. Cyclosporine may be considered a possible treatment for EC, particularly in cases unresponsive to conventional treatments or with suspected immune‐mediated pathogenesis. However, further studies and reports of success are needed to establish its efficacy and confirm its use for these conditions. Although a favorable clinical response was observed in the present case, extrapolation of this therapeutic approach to other dogs with EC should be made cautiously, given the limited veterinary evidence currently available. In the authors′ opinion, cyclosporine may be considered primarily in dogs with histopathologically confirmed EC, particularly in refractory cases unresponsive to conventional therapy, in patients with suspected immune‐mediated disease, or when prolonged corticosteroid therapy is contraindicated or undesirable.

Limitations of this case report include the evaluation of a single patient, the concomitant use of antibiotics and prior partial cystectomy, which may have influenced the clinical outcome, and the absence of serum cyclosporine level monitoring. In addition, no repeat histopathological evaluation was performed after treatment, and follow‐up was based solely on clinical improvement and ultrasonographic findings. Further studies investigating the use of cyclosporine in dogs with EC are warranted to better define its therapeutic role and causal effect.

## Author Contributions

Lara Vilela Soares: conceptualization, data curation, investigation, and writing—original and draft; Yury Carantino Costa Andrade: conceptualization, data curation, investigation, and writing—review and editing; Maria Eduarda Raffaini de Oliveira Cunha: review and editing; Alessandra Aparecida Medeiros Ronchia: visualization and writing—review and editing; Vanessa Martins Fayad Milken: visualization and writing—review and editing; Sofia Borin‐Crivellenti: supervision and writing—review and editing; Leandro Z. Crivellenti: supervision and writing—review and editing.

## Funding

This work was supported by Coordenação de Aperfeiçoamento de Pessoal de Nível Superior (10.13039/501100002322, ROR Identifier: 00x0ma614).

## Disclosure

All the authors have read and approved the published version of the manuscript.

## Ethics Statement

The animal described in this report came from a clinical appointment at a veterinary hospital; the report was written after authorization from the animal′s owner through a signed informed consent form.

## Conflicts of Interest

The authors declare no conflicts of interest.

## Data Availability

The data that support the findings of this study are openly available in [dataset]Soares. (2026). Cyclosporine as a Novel Therapeutic Option for Eosinophilic Cystitis in a Paraplegic Dog [Data set]. In Case Reports in Veterinary Medicine. Zenodo.10.5281/zenodo.18131547

## References

[1] Schmitz-Dräger B. J. , Skutetzki A. , Rieker R. J. , Schwab S. A. , Stöhr R. , Bismarck E. , Savov O. , Ebert T. , Benderska-Söder N. , and Hartmann A. , Eosinophilic Cystitis Mimicking Bladder Cancer-Considerations on the Management Based Upon a Case Report and a Review of the Literature, Virchows Archiv. (2021) 479, no. 3, 523–527, 10.1007/s00428-021-03049-x, 33576854. 33576854

[2] Forsythe P. and Paterson S. , Ciclosporin 10 Years on: Indications and Efficacy, Veterinary Record. (2014) 174, no. Supplement 2, 13–21, 10.1136/vr.102484. 24682697 PMC3995292

[3] Fuentealba I. C. and Illanes O. G. , Eosinophilic Cystitis in 3 Dogs, Canadian Veterinary Journal. (2000) 41, no. 2, 130–131, 10723601. PMC1476291 10723601

[4] Swetha K. , Reddy B. S. , and Sivajothi S. , Ultrasonographic Diagnosis of Polypoid Cystitis With Struvite Crystals in a Shih Tzu Dog, International Clinical Pathology Journal. (2023) 10, no. 1, 42–43, 10.15406/icpjl.2023.10.00217.

[5] Saadi A. , Bouzouita A. , Ayed H. , Kerkeni W. , Cherif M. , Ben Slama R. M. , Derouiche A. , and Chebil M. , Pseudotumoral Eosinophilic Cystitis, Urology Case Rreports. (2015) 3, no. 3, 65–67, 10.1016/j.eucr.2015.01.006. PMC4714248 26793503

[6] Mosholt K. S. , Dahl C. , and Azawi N. H. , Eosinophilic Cystitis: Three Cases, and a Review Over 10 Years, Case Reports. (2014) 2014, bcr2014205708, 10.1136/bcr-2014-205708, 25312971. PMC4202081 25312971

[7] Rossanese M. , Palumbo V. , Sioletic S. , Crestani A. , Giannarini G. , and Ficarra V. , Surgical Treatment of Eosinophilic Cystitis in Adults: A Report of Two Cases and a Literature Review, Urologia Internationalis. (2019) 102, no. 1, 122–124, 10.1159/000485257, 29554647. 29554647

[8] Braz L. A. N. , Cruz N. R. N. , Santos G. F. M. , Magalhães L. F. , and Crivellenti L. Z. , Eosinophilic Cystitis, a Rare Cause of Persistent Haematuria in a Diabetic Dog, Brazilian Journal of Veterinary Research and Animal Science. (2021) 58, e178389, 10.11606/issn.1678-4456.bjvras.2021.178389.

[9] Evason M. D. and Carr A. P. , Eosinophilic Cystitis in a Female German Wire-Haired Pointer, Canadian Veterinary Journal. (2007) 48, no. 5, 509–511. PMC1852600 17542370

[10] Cleva M. , Ennio B. , Ferdinando B. , and Massimo V. , Eosinophilic Cystitis: A Case Report of a Pseudotumoral Lesion, Journal of Ultrasound. (2023) 26, no. 4, 935–937, 10.1007/s40477-023-00816-1. 37589872 PMC10632338

[11] Shi G. , Wang L. , Peng G. , An X. , Lu X. , Wu H. , and Li Y. , Misdiagnosis of Eosinophilic Cystitis: A Case Report and Literature Review, Medicine. (2024) 103, no. 7, e36668, 10.1097/MD.0000000000036668, 38363913. 38363913 PMC10869055

[12] Simonato F. A. , Pavan N. , Pinelli M. , Tulone G. , Giaimo R. , Martorana A. , and Simonato A. , A Conservative Treatment for Eosinophilic Cystitis, IJU Case Reports. (2023) 6, no. 1, 8–12, 10.1002/iju5.12533, 36605690. 36605690 PMC9807346

[13] Cooke W. D. and Cooke A. J. T. , Successful Treatment of Eosinophilic Cystitis With Benralizumab, Urology Case Reports. (2020) 33, 101379, 10.1016/j.eucr.2020.101379, 33102077. 33102077 PMC7574143

[14] Chuang F. C. and Kuo H. C. , Fusion of CCL21 Non-Migratory Active Breast Epithelial and Breast Cancer Cells Give Rise to Ccl21 Migratory Active Tumor Hybrid Cell Lines, PLoS One. (2013) 8, no. 5, e63711, 10.1371/journal.pone.0063711, 23667660. 23667660 PMC3646822

[15] Archer T. M. , Boothe D. M. , Langston V. C. , Fellman C. L. , Lunsford K. V. , and Mackin A. J. , Oral Cyclosporine Treatment in Dogs: A Review of the Literature, Journal of Veterinary Internal Medicine. (2014) 28, no. 1, 1–20, 10.1111/jvim.12265. 24341787 PMC4895546

[16] Aleem S. , Kumar B. , Fasano M. B. , Takacs E. , and Azar A. E. , Successful Use of Cyclosporine as Treatment for Eosinophilic Cystitis: A Case Report, World Allergy Organization Journal. (2016) 9, no. 9, 10.1186/s40413-016-0113-4, 27458500. PMC4939020 27458500

[17] Walker M. A. and Cole G. , Jejunal Perforation and Septic Abdomen Resulting From a Choristoma in a Dog, Canadian Veterinary Journal. (2024) 65, no. 1, 29–32, 38164377. PMC10727160 38164377

[18] Tofte N. , Nielsen A. C. Y. , Trøstrup H. , Andersen C. B. , Von Linstow M. , Hansen B. , Biering-Sørensen F. , Høiby N. , and Moser C. , Chronic Urinary Tract Infections in Patients With Spinal Cord Lesions – Biofilm Infection With Need for Long-Term Antibiotic Treatment, Apmis. (2017) 125, no. 4, 385–391, 10.1111/apm.12685, 28407430. 28407430

[19] Dubucquoi S. , Janin A. , Desreumaux P. , Rigot J. M. , Copin M. C. , François M. , Torpier G. , Capron M. , and Gosselin B. , Evidence for Eosinophil Activation in Eosinophilic Cystitis, European Urology. (1994) 25, no. 3, 254–258, 10.1159/000475293. 8200410

[20] Kullmann F. A. , Clayton D. R. , Ruiz W. G. , Wolf-Johnston A. , Gauthier C. , Kanai A. , Birder L. A. , and Apodaca G. , Urothelial Proliferation and Regeneration After Spinal Cord Injury, American Journal of Physiology-Renal Physiology. (2017) 313, no. 1, F85–F102, 10.1152/ajprenal.00592.2016, 28331065. 28331065 PMC5538841

[21] Janzen J. , Bersch U. , Pietsch-Breitfeld B. , Pressler H. , Michel D. , and Bültmann B. , Urinary Bladder Biopsies in Spinal Cord Injured Patients, Spinal Cord. (2001) 39, no. 11, 568–570, 10.1038/sj.sc.3101218. 11641805

[22] Wu S. Y. , Jiang Y. H. , Jhang J. F. , Hsu Y. H. , Ho H. C. , and Kuo H. C. , Inflammation and Barrier Function Deficits in the Bladder Urothelium of Patients With Chronic Spinal Cord Injury and Recurrent Urinary Tract Infections, Biomedicines. (2022) 10, no. 2, 10.3390/biomedicines10020220, 35203430. PMC8868780 35203430

[23] Chen Y. , Ji M. , Shi J. , Qiao Z. , Tang L. , and Yang Z. , The Clinical and Imaging Features of Eosinophilic Cystitis in Children: A Case Series Study, International Urology and Nephrology. (2024) 56, no. 2, 363–371, 10.1007/s11255-023-03811-x. 37782435

[24] Salman M. , Al-Ansari A. A. , Talib R. A. , Malik E. F. , Al-Bozaom I. A. , and Shokeir A. A. , Eosinophilic Cystitis Simulating Invasive Bladder Cancer: A Real Diagnostic Challenge, International Urology and Nephrology. (2006) 38, no. 3-4, 545–548, 10.1007/s11255-006-0103-5, 17115289. 17115289

[25] Hasan A. , Abdel-Al I. , Nafie K. , Rashad M. F. , Abozied H. , Elhussiny M. E. A. , Rabie A. , Rabaan A. A. , Abd Elnabi M. K. , Abdelwahed M. S. , Ahmed M. A. , and Mohammed Y. , Clinicopathological Characteristics and Outcomes of Eosinophilic Cystitis: A Retrospective Study, Annals of Medicine and Surgery. (2021) 68, 102626, 10.1016/j.amsu.2021.102626, 34386222. 34386222 PMC8346616

[26] Van den Ouden D. , Eosinofiele Cystitis, Tijdschrift voor Urologie. (2023) 13, no. 2-3, 41–43, 10.1007/s13629-023-00388-z.

[27] Adeleye O. , Trickett J. S. , Wright B. L. , and Khan A. , Refractory Eosinophilic Cystitis Controlled With Low-Dose Cyclosporine Therapy: A Case Report, Urology Case Reports. (2021) 39, 101829, 10.1016/j.eucr.2021.101829, 34522620. 34522620 PMC8424508

